# Reducing Negative Attitudes Toward Immigrants in Russia and Taiwan: Possible Beneficial Effects of Naïve Dialecticism and an Incremental Worldview

**DOI:** 10.3389/fpsyg.2020.572386

**Published:** 2020-09-11

**Authors:** I-Ching Lee, Tatyana Permyakova, Marina Sheveleva

**Affiliations:** ^1^Department of Psychology, National Taiwan University, Taipei, Taiwan; ^2^Department of Foreign Languages, National Research University Higher School of Economics, Perm, Russia

**Keywords:** intergroup relations, social dominance orientation, right-wing authoritarianism, cross-cultural comparison, model testing

## Abstract

Greater mobility in human societies has resulted in more interactions and contact with immigrants. In the current research, we investigated how viewing the world as flexible, changing, and paradoxical (i.e., naïve dialecticism and an incremental theory) may predict one’s authoritarian beliefs and in turn predict one’s attitudes toward immigrants. To test the generalizability of our findings, we recruit comparable samples (i.e., college students) from two societies that are largely different (Russia and Taiwan). Great cultural similarities were observed. Naïve dialecticism and an incremental theory appeared as two distinctive constructs. People who were higher on naïve dialecticism and an incremental over entity theory had lower support for authoritarian beliefs (i.e., right-wing authoritarianism and social dominance orientation) and, in turn, had more favorable attitudes toward immigrants. Some cultural differences were also observed. Taiwanese participants’ negative attitudes toward immigrants were entirely ideology-based, whereas Russian participants’ negative attitudes toward immigrants were partly based on presumably personal experiences. Pan-cultural and culturally specific mechanisms in predicting attitudes toward immigrants were further discussed and explored.

## Introduction

Culture provides an interpretive framework for individuals to make sense of the world, such as through cultural values, social norms, and lay beliefs. How individuals endorse these values, social norms, and lay beliefs has a profound impact on their relations with others. In our research, we investigate two broad types of lay beliefs (i.e., naïve dialecticism, Peng and Nisbett, 1999; Spencer-Rodgers et al., 2010, and entity vs. incremental theories, Dweck et al., 1995) and how they may have impacts on people’s attitudes toward immigrants.

Naïve dialecticism is commonly observed among East Asians (Peng and Nisbett, 1999; Spencer-Rodgers et al., 2010). The long history of dialectical reasoning could be traced to Marx and Engels in philosophy (Peng and Nisbett, 1999) and Taoist, Buddhist, and Confucian epistemologies (Spencer-Rodgers et al., 2010). Naïve dialecticism denotes the back-and-forth debate between opposing sides and includes several essential elements (Peng and Nisbett, 1999; Spencer-Rodgers et al., 2012): (a) the concept of change (reality is not fixed or static, but an ongoing process); (b) the concept of contradiction (reality is paradoxical), and (c) the concept of holism (nothing is isolated). Previous studies have found that East Asians are more likely to exhibit naïve dialecticism than Americans and Europeans (e.g., Chinese, Peng and Nisbett, 1999). Eastern Europeans, including Russians, are found to be more holistic in terms of categorization and visual perception, a characteristic of naïve dialecticism, than Western Europeans (Varnum et al., 2008).

In addition to naïve dialecticism, a similar construct has been proposed, entity vs. incremental theories. Similar to naïve dialecticism, an incremental theory is to see human actions and outcomes not as fixed, but as dynamic, malleable, and developable (Dweck et al., 1995). People with such lay beliefs are called incremental theorists, or incrementalists, in comparison to those who see human actions and outcomes as fixed and non-malleable, who are known as entity theorists (Dweck et al., 1995). Both naïve dialecticism and entity vs. incremental theories have been linked with intergroup relations (e.g., Carr et al., 2012 for a review of entity vs. incremental theories on intergroup relations; Spencer-Rodgers et al., 2012 for a review of naïve dialecticism on intergroup relations). Naïve dialecticism and incremental vs. entity theories are developed and studied by two separate groups of researchers (e.g., Kaiping Peng, Julie Spencer-Rodgers on the one hand, and Carol S. Dweck, Sheri R. Levey on the other hand), and, to our best knowledge, no study has examined the similarities and differences between the two. The lack of contrast and comparison between naïve dialecticism and incremental vs. entity theories may raise questions with regard to discriminant validity and unique contributions of either construct. Thus, the first purpose of our research is to investigate the similarities and differences between naïve dialecticism and incremental vs. entity theories.

To test the unique contributions of naïve dialecticism and incremental vs. entity theories on intergroup relations, we study individuals’ negative attitudes toward immigrants. Because dialectical thinkers recognize both the good and bad aspects of immigrants, dialectical thinkers may hold less negative attitudes toward immigrants than non-dialectical thinkers. Consistent evidences have been observed in that dialectical thinkers express lower ingroup favoritism (Ma-Kellams et al., 2011) and use less group stereotypes (Spencer-Rodgers et al., 2012) than non-dialectical thinkers. Chang and Chiou (2014) found that the more Taiwanese participants endorse naive dialecticism, the more they endorse multiculturalism, a belief which recognizes and celebrates group differences, and reject color blindness, a belief which minimizes group differences and preserves the privileges of the dominant groups. Similarly, because entity [incremental] theorists view individual characteristics and relationships as fixed [malleable], they are more [less] likely to stereotype (Rydell et al., 2007) and avoid [attempt to resolve] potential conflicts (Carr et al., 2012) and may have higher [lower] negative attitudes toward immigrants.

To investigate independent effects of naïve dialecticism and incremental vs. entity theorists on negative attitudes toward immigrants, however, two robust perceivers’ characteristics constantly identified in the literature need to be considered: right-wing authoritarianism (RWA) and social dominance orientation (SDO, e.g., in a meta-analysis, Cohrs and Stelzl, 2010). Right-wing authoritarians are anxiety-ridden and preoccupied with the distinction of power and weakness (Adorno et al., 1950). Conversely, a tendency to support a conventional hierarchical society is called social dominance orientation (SDO, Sidanius and Pratto, 1999). Many researchers have relied on Duckitt’s (2006) Dual Process Cognitive-Motivational Theory to distinguish between and understand the effects of RWA and SDO on how we view people who do not belong to our groups (i.e., outgroups). Duckitt (2006) argued that there are two motivational mechanisms. High RWA individuals may view outgroups as threats to social control, order, and stability (a dangerous world) and view them negatively. High SDO individuals may view outgroups as competitors in regard to struggles over relative dominance and superiority (a competitive jungle world) and view them negatively. Supporting the model, Duckitt and Sibley (2010) found that high RWA individuals are more likely to oppose certain migrants in New Zealand (e.g., Sandrian) when thinking about their economic competition and social threat, whereas high SDO individuals are more likely to oppose these migrants when thinking about their economic competition and their disadvantaged group status.

In the current research, we investigate how lay beliefs (i.e., naïve dialecticism, incremental vs. entity theorists) may predict authoritarian beliefs (i.e., RWA orientation and SDO) and in turn individuals’ attitudes toward immigrants. Our approach is different from previous researchers’ approaches using stable individual differences (e.g., Big Five, Ekehammar et al., 2004) or group positions (e.g., Guimond et al., 2003) to explain the associations between authoritarian beliefs and views of outgroup members. For example, Ekehammar et al. (2004) found that individuals low on openness to experience (agreeableness) would be high on RWA (SDO) and in turn would show generalized prejudice. In addition, Guimond et al. (2003) showed that dominant group members are more likely to support SDO and show negative attitudes toward immigrants than subordinate group members. Although both approaches provide insight to the associations between authoritarian beliefs and attitudes toward immigrants, personality characteristics and group positions are relatively stable. Lay beliefs, however, have been identified to vary across situations and could be experimentally manipulated (e.g., Carr et al., 2012). If the lay beliefs may reduce authoritarian beliefs and negative attitudes toward immigrants, the changes in the lay beliefs may have important consequences.

To the best of our knowledge, the examination of how lay beliefs may predict authoritarian beliefs and in turn individuals’ attitudes toward immigrants has never been done. Thus, we conduct this research in two non-Western countries, Taiwan and Russia, because both countries are relatively understudied with regard to this topic and they differ economically, culturally, and in terms of national power. Russia is the largest country by surface area in the world and has a GDP per capita of 26,100 international dollars. There are approximately one hundred and forty-seven million people in Russia, largely consisting of Russians (approximately 81%). Taiwan, on the other hand, consists of islands, is barely recognized as a sovereign state in the international context, and has a GDP per capita of 47,800 international dollars. There are approximately twenty-three million people in Taiwan, largely consisting of Han Taiwanese (approximately 95%).

Due to the large socioeconomic and historical differences in the two countries, cross-cultural similarities may attest to the potential cross-cultural universality of the underlying mechanisms, whereas cross-cultural differences may inform us of culturally specific mechanisms. On the one hand, because immigrants are often considered outsiders in different countries, we expect that lay beliefs and authoritarian beliefs would be linked with negative attitudes toward immigrants in both societies. On the other hand, if immigrants are negatively evaluated due to the history and status in the given society, the reasons for why people hold negative attitudes toward immigrants may differ across societies. For example, due to the relative racial compositions of the populations (81% Russians vs. 95% Han Taiwanese) and the relative sizes of immigrants and migrants (8.0% immigrants and 7.2% migrants in Russia in 2019, United Nations, 2019, vs. 0.24% immigrants and 0.18% migrants in Taiwan, Department of Household Registration, and Ministry of the Interior, Taiwan, 2020), residents in Russia are more likely to encounter immigrants or have relatives as migrants, in comparison to the residents in Taiwan. If the actual experiences related to immigration transform or challenge one’s attitudes toward immigrants, we expect that there should be some cultural difference in that those who have relatives as migrants may hold less negative attitudes toward immigrants than those who do not have (path e). We explored to see whether the association may differ in the two cultural contexts.

The inclusion of the two non-western societies further allows us to test whether naïve dialecticism and entity vs. incremental theorists may be linked with authoritarian beliefs and attitudes toward immigrants. Based on the findings that people in Taiwan and Russia generally understand and support naïve dialecticism more than Americans or Western Europeans (e.g., Chinese, Peng and Nisbett, 1999; Russians, Varnum et al., 2008), we tested mixed findings in Kahn et al. (2018). Kahn et al. (2018) tested how incremental vs. entity theories, RWA, and SDO may predict one’s political identity (liberal vs. conservative) in the United States, Sweden, and Israel. They found that the more one endorses the entity view of groups, the more one endorses SDO and as a result supports a conservative political identity in the United States and Sweden. In other words, SDO mediates the relationship between entity vs. incremental theories on groups and political identity. However, Kahn and colleagues neither observed a mediation effect of RWA in the United States, Sweden, or Israel nor observed a mediation effect of SDO in Israel. Because the liberal–conservative political identity may be construed differently in the respective societies (e.g., Duriez et al., 2005), it is not clear whether the divergent findings are due to the different conceptualizations of liberal–conservative political identity, or that the associations between lay beliefs and authoritarian beliefs actually differ in these societies. Thus, our second purpose of the research is to test cross-cultural similarities and differences in the associations between lay beliefs, authoritarian beliefs, and negative attitudes toward immigrants.

**FIGURE 1:**
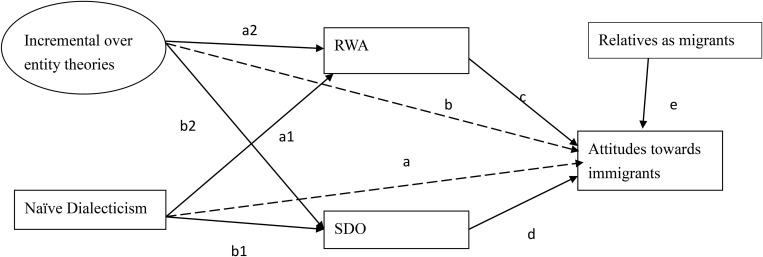
A model testing the associations between an incremental over entity theories, naïve dialecticism, right-wing authoritarianism, social dominance orientation, and negative attitudes toward immigrants. Participants’ age was controlled for but not shown (see the results presented in Table 2). The dotted lines indicate non-significant associations in the final model. RWA, right-wing authoritarianism; SDO, social dominance orientation.

To recapitulate, in the current study, we examined how naïve dialecticism, an incremental over entity theory, RWA, and SDO may predict individuals’ attitudes toward immigrants in Russia and Taiwan. Because naïve dialecticism and incremental vs. entity theories are broader in content, we expected that they would be distal causes, whose effects on negative attitudes toward immigrants were mediated by authoritarian beliefs (i.e., RWA and SDO). In short, we tested the model presented in Figure 1 (and also the final model) in which naïve dialecticism and an incremental view of the world are proposed to reduce RWA and SDO (paths a and b) and, in turn, RWA and SDO are proposed to increase negative attitudes toward immigrants (paths c and d). We also tested whether individuals’ relatives as migrants may predict their attitudes toward immigrants (path e). The paths were tested to determine whether they could be fixed in the two cultural contexts to reflect cultural similarities.

## Materials and Methods

### Participants

To ensure the comparability of the two samples, we recruited Taiwanese and Russian nationals currently studying at universities in Taipei, Taiwan, and Perm, Russia. In Taiwan, we posted our ads in the Department of Psychology, National Taiwan University, as well as the school’s online forums frequented by college students. In Russia, we posted our ads at National Research University Higher School of Economics. There were 198 Taiwanese respondents (142 females, one gender unknown) and 199 Russian respondents (109 females). On average, these respondents were aged 19.9 (in Taiwan) and 19.6 years (in Russia), and 32.8% of the Taiwanese (19.0% Russian) respondents reported having relatives living abroad or having a different nationality. We should note that our sample size in each culture is considered acceptable according to a minimum sample of a case-per-indicator ratio of 5–10 (Wang and Wang, 2012, our ratio is 20), or a general rule of thumb (about 200 cases in a model, Kelloway, 1998). All procedures were in accordance with the ethical standards of the institutional research committee and with the 1964 Helsinki Declaration and its later amendments or comparable ethical standards. Following all the research ethic codes of respective universities, the Ethical Review Approval was issued by National Taiwan University (NTU-REC No.: 201805HS002), the institute of the first author who initiated the study. The study procedures in Russia complied with the signed research ethical guidelines of National Research University Higher School of Economics.

### Measures

All respondents read the information consent before filling out a questionnaire; the process took about 20–30 min. All materials were first prepared in English but presented in the official language of the respective country. These materials were either translated by previous researchers or translated by the research team following Brislin’s (1970) three-step back-translation. In the process, a native bilingual speaker first translated the materials into Chinese (in Taiwan) or Russian (in Russia). Then, a separate bilingual speaker back-translated the materials into English. Third, the research team compared the two versions for accuracy and clarity and made adjustments accordingly. Participants responded to the following measures using a 1–6 Likert-type scale, unless otherwise noted.

#### Dialectical Self Scale (DSS, Spencer-Rodgers et al., 2015, 32 Items)

One example item for the dialectical self scale was “I often change the way I am, depending on who I am with.” Confirmatory factor analysis was performed to identify and exclude problematic items. A short version of the original scale reached a satisfactory factor structure (10 items, see the Appendix), setting the paths and covariances to be the same in the two cultures, with fitting indices of χ^2^(df = 74) = 89.30, *p* = 0.11, CFI = 0.94, RMSEA = 0.023 (0.000,0.038). The higher the score, the more dialectical self-view one has.

#### Implicit Theory Scale on the Worldview

The scale was measured using three items (Dweck et al., 1995). One example item for the worldview was “Although some phenomena can be changed, it is unlikely that the core dispositions of the world can be altered.” The higher the score, the more incremental (over entity) view a person has. The three items all converged on the factor, but the paths were not the same in the two cultures. The structural equivalent model had fitting indices of χ^2^(df = 1) = 2.03, *p* = 0.15, CFI = 1.00, RMSEA = 0.051 (0.000,0.154).

#### Social Dominance Orientation (Pratto et al., 2000; Ho et al., 2015)

Twelve items were selected from Pratto et al. (2000) and Ho et al. (2015). An example item was “Some groups of people are simply inferior to other groups.” A shorter version of the original scale reached a satisfactory factor structure (8 items, see the Appendix), setting the paths and covariances to be the same in the two cultures, with fitting indices of χ^2^(df = 43) = 80.26, χ^2^/df < 2, CFI = 0.97, RMSEA = 0.047 (0.031,0.063). The higher the score, the more socially dominant one’s view is.

#### Right-Wing Authoritarianism (Duckitt et al., 2010)

Fifteen items were selected from Duckitt et al. (2010). An example item was “Our country will be great if we show respect for authority and obey our leaders.” A shorter version of the original scale reached a satisfactory factor structure (12 items, three sub-factors, see the Appendix), setting the paths and covariances to be the same in the two cultures, with fitting indices of χ^2^(df = 114) = 237.30, χ^2^/df < 2.2, CFI = 0.95, RMSEA = 0.052 (0.043,0.062). The higher the score, the more RWA one believes.

*Attitudes toward immigrants* were measured by using a relevant section in the International Social Survey Program (the ISSP 2013). An example of a statement was “Immigrants take away jobs from people who were born in [X].” Because exploratory factor analysis showed that the items loaded on the same factor, Eigen value = 3.27, factor loadings > 0.45, we did not further distinguish threat perceptions and general attitudes. Seven items assessing realistic threats (three items; jobs, economy, security), symbolic threats (two items; culture and ideas), and general attitude (enjoying equal rights, exclusion) converged on a satisfactory factor structure, setting the paths and covariances to be the same in the two cultures, with fitting indices of χ^2^(df = 32) = 55.16, χ^2^/df < 2, CFI = 0.96, RMSEA = 0.043 (0.023,0.062). Due to the different scales used in various items, items were standardized before being averaged. The higher the score, the more negative attitude one has toward immigrants.

#### Background Information

Respondents were asked to indicate their age, gender, citizenship, education, and place of birth, as well as whether they have close relatives who do not have Russian/Taiwanese citizenship or constantly living abroad (yes or no).

## Results

We first investigated cultural and gender differences in the five variables (naïve dialecticism, incremental vs. entity theories, SDO, RWA, and negative attitudes toward immigrants). Based on a two-way multivariable analysis of covariance with culture and sex as independent variables, controlling for two covariates (age, migrant relatives), there were robust cultural differences in naïve dialecticism, SDO, RWA, and negative attitudes toward immigrants, in which Taiwanese endorse more naïve dialecticism (on a scale of 1–6, *M*_T_ = 4.48 vs. *M*_R_ = 4.06, *p* < 0.001, ρ^2^ = 0.13, see Table 1) than Russians, whereas Russians endorse more SDO (*M*_R_ = 3.09 vs. *M*_T_ = 2.43, *p* < 0.001, ρ^2^ = 0.11), RWA (*M*_R_ = 2.83 vs. *M*_T_ = 2.57, *p* = 0.002, ρ^2^ = 0.02), and negative attitudes toward immigrants (*M*_R_ = 3.49 vs. *M*_T_ = 2.66, *p* < 0.001, ρ^2^ = 0.22) than Taiwanese. However, the two groups did not differ with regard to incremental vs. entity theories (*M*_D_ = −0.04, *p* = 0.73). The above findings suggest that naïve dialecticism and the implicit theory of the worldview are distinctive constructs.

**TABLE 1:** Means and confidence intervals on naïve dialecticism, incremental theories of worldview, authoritarian beliefs, and attitudes toward immigrants.

	Russia			Taiwan			Gender comparisons	
	Men	Women	Total	Men	Women	Total	Men	Women
Naïve dialecticism^a,1,2^	3.98 (3.87, 4.09)	4.14 (4.05, 4.24)	4.06 (3.99, 4.13)	4.38 (4.25, 4.52)	4.57 (4.48, 4.65)	4.48 (4.40, 4.56)	4.18 (4.09, 4.27)	4.36 (4.29, 4.42)
IT of the worldview^a,b,3^	3.64 (3.43, 3.84)	4.11 (3.92, 4.30)	3.87 (3.74, 4.01)	4.00 (3.73, 4.26)	3.68 (3.52, 3.84)	3.84 (3.68, 3.99)	3.82 (3.65, 3.98)	3.90 (3.77, 4.02)
SDO^a,c,1,2^	3.16 (2.98, 3.34)	3.01 (2.84, 3.18)	3.09 (2.96, 3.21)	2.62 (2.38, 2.85)	2.25 (2.11, 2.39)	2.73 (2.30, 2.57)	2.89 (2.74, 3.04)	2.63 (2.52, 2.74)
RWA^a,d,1,3^	2.71 (2.54, 2.88)	2.95 (2.80, 3.11)	2.83 (2.72, 2.94)	2.69 (2.47, 2.90)	2.44 (2.31, 2.57)	2.57 (2.44, 2.69)	2.70 (2.56, 2.84)	2.70 (2.60, 2.80)
Negative attitudes toward immigrants (ATI)^e,1^	0.39 (0.27, 0.52)	0.29 (0.18, 0.40)	0.34 (0.26, 0.43)	−0.40 (−0.56, −0.24)	−0.30 (−0.40, −0.21)	−0.35 (−0.44, −0.26)	−0.002 (−0.10, 0.10)	−0.01 (−0.08, 0.07)
Sample size	90	105	195	55	142	197	145	247

*^*a*^On a scale of 1–6. ^*b*^Implicit theories of the world (incremental vs. entity), ^*c*^social dominance orientation, ^*d*^right-wing authoritarianism, ^*e*^due to different scales used in the items, items were standardized and averaged. ^1^Cultural difference, ^2^Gender difference, ^3^Interaction between culture and gender. A multivariate analysis of covariance (MANCOVA) was conducted with two independent variables (country and gender) and two covariates (age and relatives as migrants).*

The evidence that naïve dialecticism and the implicit theory of the worldview are conceptually distinctive could also be corroborated in the gender comparison. Women demonstrated more naïve dialecticism than men (*M* = 4.36 vs. *M* = 4.18, *p* = 0.002, ρ^2^ = 0.024), but they did not differ from men on the incremental vs. entity theories (*M*_D_ = 0.08, *p* = 0.47). In addition, in none of the subsamples (Taiwanese men and women, Russian men and women) were the correlations of naïve dialecticism and the implicit theory of the worldview significant (all other comparisons, *r*s < 0.11, *p*s > 0.22).

We further investigated whether naïve dialecticism and the implicit worldview may account for the support of authoritarian beliefs (i.e., RWA, SDO) and negative attitudes toward immigrants in the two cultural samples (see Figure 1), as shown in the means and confidence intervals presented in Table 1 and regression coefficients presented in Table 2.

**TABLE 2:** Naïve dialecticism and the incremental worldview on the negative attitudes toward immigrants and authoritarian beliefs: unstandardized coefficients and standard errors.

Paths	TW	RUS
Incremental theories of the worldRWA (path a1)	−0.23(0.05)***	
Naïve dialecticismRWA (path a2)	−0.21(0.08)**	
Incremental theories of the world SDO (path b1)	−0.12(0.05)*	
Naïve dialecticismSDO (path b2)	−0.15(0.08) +	
RWANATI (path c)	0.18(0.03)***	
SDO NATI (path d)	0.15(0.03)***	
Relatives as migrants NATI (path e)	−0.01(0.06)	−0.28(0.12)*
Age NATI	0.01 (0.02)	0.13(0.04)**

RWA, right-wing authoritarianism; SDO, social dominance orientation. NATI, negative attitudes toward immigrants. ^+^*p* < 0.10; * *p* < 0.05; ** *p* < 0.01; *** *p* < 0.001.

We conducted model testing with a bootstrapping method of 2,000 resamples. Because there was neither gender effect nor an interaction effect between culture and gender on negative attitudes toward immigrants (see Table 1 for the means and confidence intervals), we tested the model regardless of one’s gender. Because naïve dialecticism, authoritarian beliefs, and attitudes toward immigrants display cultural equivalence (same items, same loadings), to simplify the model, we calculated the means of the above variables. Because the loadings of the implicit worldview items vary according to cultures, we incorporated a latent variable in the model and allowed for the loadings to vary across cultures. Using a multigroup method, each path was fixed to test whether it could be the same across cultures. If the change of chi-square values significantly increased, fixing the path would not be considered appropriate. In the final model, we are able to fix six paths (out of eight paths) and one covariance (out of two covariances) to be the same in Russia and Taiwan, and the model had acceptable fitting indices, χ^2^(df = 53) = 45.45, *p* = 0.76, CFI = 1.00, RMSEA = 0.000 (0.000,0.023). The results showed that there were large cultural similarities and a few cultural differences.

First, naïve dialecticism and incremental vs. entity theories had independent negative associations with RWA and SDO in both cultures. The more individuals hold naïve dialecticism and an incremental over entity theory, the less they support RWA and SDO. Moreover, RWA and SDO were associated with negative attitudes toward immigrants, and these associations were similar in magnitude in the two cultures. The indirect effect of naïve dialecticism on negative attitudes toward immigrants via SDO and RWA was significant −0.06 [−0.10, −0.02], *p* = 0.005, whereas the indirect effect of an incremental theory on negative attitudes toward immigrants via SDO and RWA was also significant, −0.06 [−0.10, −0.04], *p* = 0.001. We should note that after controlling for all the other variables, naïve dialecticism and an incremental view of the world were actually negatively correlated, albeit very weakly (*r* = −0.05, *p* = 0.02), in both cultural contexts.

Nevertheless, there were a few cultural differences. First, participants’ relatives as migrants predicted their attitudes toward immigrants in Russia (see the bottom second row in Table 2) but not in Taiwan. Having migrant relatives mitigated the negative attitudes they hold toward immigrants. In addition, participants’ age predicted their attitudes toward immigrants in Russia (see the bottom row in Table 2). The older the individuals in Russia, the more negative attitudes they hold toward immigrants. Furthermore, the covariance between SDO and RWA was significant and moderate in Taiwan (*r* = 0.32, *p* < 0.001) but marginal in Russia (*r* = 0.09, *p* = 0.052).

Testing an alternative model. We also tested whether an alternative model may fit the data well by reversing the proposed causal effect of lay beliefs on authoritarian beliefs. That is, authoritarian beliefs were treated as distal causes, naïve dialecticism and incremental over entity theories as mediators, and negative attitudes toward immigrants as the outcome. The alternative model was considerably poorer than the proposed model, χ^2^(df = 53) = 109.58, *p* < 0.001, CFI = 0.92, RMSEA = 0.052 (0.038,0.066). Moreover, the reversed paths from social dominant orientation to naïve dialecticism and incremental vs. entity theories were not significant (*r*s < −0.05, *p*s > 0.42), nor was the path from RWA to naïve dialecticism (*r* = −0.06, *p* = 0.10).

## Discussion

We adopted an etic approach to explore (1) the similarities and differences of naïve dialecticism and incremental vs. entity theorists and (2) the potential effects of naïve dialecticism and incremental vs. entity theorists on attitudes toward immigrants via authoritarian beliefs. We must first acknowledge its limitations. First, to adopt an etic approach in our research, we could not examine how people may hold attitudes toward different immigrant groups but instead targeted general attitudes toward immigrants. Immigrants form heterogeneous groups, however, and people may hold different views toward these different groups. Future researchers should closely compare and contrast these immigrant groups to offer some insight into understanding the relationships between domestic people and immigrants. Second, previous researchers have distinguished threat perceptions and used such perceptions to predict attitudes toward immigrants (e.g., Stephan et al., 1999). Because the exploratory factor analysis in our sample shows that these seven items loaded on the same factor, we did not further separate threat perceptions and general attitudes toward immigrants. Third, to have comparable samples in Russia and Taiwan, we targeted college students to control for educational level, age, and economic status. It remains to be seen, however, whether the largely culturally similar findings could be replicated among people of different backgrounds (e.g., older individuals, people with lower education).

In the literature, naïve dialecticism and incremental vs. entity theorists are studied by different groups of researchers, although both have been proposed to be associated with intergroup relations. In our research, because the two constructs are not positively correlated (non-significant raw correlations in the four subsamples and a significant but negative partial correlation in both Russia and Taiwan in the model), we showed that naïve dialecticism and incremental vs. entity theorists are distinctive constructs, despite some similarities in the definitions (e.g., an incremental theory is to see the world as malleable and dynamic, Dweck et al., 1995; a component of naïve dialecticism is to view reality not as fixed or static, but an ongoing process, Spencer-Rodgers et al., 2012). Perusing the items of the culturally equivalent short scale of naïve dialecticism, at least three items were loaded on each of the three factors, suggesting the appropriate factor structure of the scale (the concepts of change, contradiction, and holism). The incremental worldview, however, was not culturally equivalent in terms of how the three items loaded on the general factor. Russians had higher loadings on two items (core dispositions of the world can be altered and little can be done to change it) than Taiwanese; each path fixed significantly increased the chi-square value, Δχ^2^_(df__=__1__)_ > 13.22, *p* < 0.001.

In addition, Taiwanese and women tend to support more naïve dialecticism than Russians and men, although there are no such cultural or gender differences in regard to the incremental theories. Instead, the incremental theories seem to be context-situated according to two pieces of evidence. First, the relative weights on the items of the implicit theories of the world differ for Russians and Taiwanese, and no cultural equivalence of the scale is achieved. Second, Russian men supported the incremental theories more than Russian women, whereas Taiwanese women supported the incremental theories more than Taiwanese men, suggesting that men and women may formulate the incremental theories differently in the two societies. The findings suggest a need to further understand how implicit theories and naïve dialecticism are developed. Previous researchers suggested that primary caretakers’ responses to children’s performance may be a key to their development of incremental over entity theories (e.g., maternal personal praise leading to children’s later entity theories of intelligence, Pomerantz and Kempner, 2013; perceived parental criticism associated with lower incremental theories of intelligence, Gunderson et al., 2018), but some cultural differences were observed (e.g., maternal psychological control was found to associated *positively* with children’s incremental theories of intelligence in three Chinese samples, Kim et al., 2017). We were not able to find any research explaining the development of naïve dialecticism. Future researchers should continue to explore the separate roots of naïve dialecticism and the incremental worldview.

Naïve dialecticism and incremental worldview act as distal causes that may reduce individuals’ support of RWA and SDO, and similar findings were observed in both societies. Previous researchers have found RWA and SDO to be relatively stable over time (e.g., βs > 0.66 after 1 year, Sibley and Duckitt, 2010) and difficult to be changed by contexts manipulated in experiments (e.g., no changes after participating in a social organization that promotes citizen education in Imhoff and Brussino, 2019; no changes after evaluating religious groups or same-sex partners benefited from federal funds in Lehmiller and Schmitt, 2007). Even when contexts showed changes in authoritarian beliefs, the effects were not robust on both SDO and RWA. Lehmiller and Schmitt found that reading aggression initiated by different targets (U.S. or Saddam) only had a marginal effect on SDO but had no effect on RWA. Engaging in Alternative Political Socialization Programme had no effect on SDO but had a significant effect on RWA (Imhoff and Brussino, 2019). From the above findings, it could be concluded that researchers have not yet developed a paradigm to experimentally change authoritarian beliefs in a short period of time. Naïve dialecticism and incremental worldview may be one way to change such beliefs.

The culturally similar findings in our research are rather imposing considering the large differences between Russia and Taiwan, in terms of the political and economic systems, ethnic composition, and national power (military power, the scope of territory). We targeted these two societies to reveal mechanisms that may be universal and culturally specific in predicting attitudes toward immigrants. The two mechanisms driven by RWA and SDO on attitudes toward immigrants are largely similar in Russia and Taiwan. The findings are in direct contrast with those of McFarland et al. (1996) and Kahn et al. (2018). Perhaps culturally similar findings could be observed only when the constructs were culturally equivalent [e.g., our adoption of culturally equivalent constructs in comparison to Kahn et al. (2018) who did not test for culturally equivalent scales]. As for the divergent findings with McFarland et al. (1996), there are two potential explanations for this difference. First, it is possible that the establishment of the Soviet Union fundamentally changed Russians’ view of the power structure in groups and that the impact gradually disappeared after the collapse of the system. Second, McFarland et al. (1996) findings may only apply to the communism and ingroup minority groups (e.g., the poor) but not the clear outsider group (i.e., the immigrants). We suspect that the latter explanation may be more potent because of the positive correlation between age and negative attitudes toward immigrants in Russia. Our findings suggest that individuals who see the world as dangerous or competitive may exclude outsiders and hold negative attitudes toward immigrants, consistent with Grigoryev and van de Vijver’s (2018) findings. We should note that according to our model, the included variables could not fully account for the cultural difference in the negative attitudes toward immigrants. Because the model is equivalent in both cultural contexts and the effects of naïve dialecticism and an incremental theory were fully mediated by authoritarian beliefs (SDO and RWA), we could estimate how much the cultural differences in RWA (*M*_D_ = 0.27) and SDO (*M*_D_ = 0.65) could account for the negative attitudes toward immigrants (*M*_D_ = 0.69). Other things being equal, the cultural differences of the authoritarian beliefs could account for a difference score of 0.15 (0.27 × 0.18 + 0.65 × 0.15 = 0.15). This finding suggests other potential mechanisms in determining one’s negative attitudes toward immigrants, which may not be captured by individuals’ authoritarian beliefs.

Although the culturally similar findings are quite compelling, we also found some cultural differences, suggesting some culturally specific mechanisms. On the one hand, the attitude of Taiwanese toward immigrants seems to be purely ideology-based; the associations between RWA and SDO, along with their attitudes toward immigrants, are all significant. Conversely, Russians’ attitudes toward immigrants seem at least partially based on personal experiences, in which the associations between a personal background, such as one’s age and having relatives as migrants, significantly predict their attitudes toward immigrants (no such associations were observed in Taiwan). These findings suggest that the composition of the society may also provide a source of information for individuals to modify attitudes toward immigrants. In a more homogenous society such as Taiwan (over 95% of the population being Han Taiwanese), its members may have very little actual experience in interacting with immigrants and their attitudes toward the immigrants are largely based on ideology. Russians, however, are a relatively diverse society, and in line with the current literature (e.g., Lebedeva and Tatarko, 2013), its members may have very different experiences with immigrants, because of the collapse of the Soviet Union and economic downturn in Central Asian countries of the former Soviet Union. Older Russians, bearing more conservative values, may form negative attitudes toward immigrants because of the intergroup conflicts that occurred earlier in Russian history (Lebedeva and Tatarko, 2013). Conversely, the collapse of the Soviet Union divided families due to the redrawn borders; such experiences may allow individuals to hold a more sympathetic view of immigrants.

Furthermore, the differences between Russia and Taiwan could explain why the Russian participants supported authoritarian beliefs more than the Taiwanese participants. Russia is one of the main powers in the international context, whereas Taiwan is a country often dominated by China (Malle, 2017). Consistent with previous research that shows that members of dominant groups tend to support authoritarian beliefs more than members of subordinate groups (e.g., SDO in a meta-analysis, Lee et al., 2011; RWA in gender and racial comparisons, Whitley et al., 2011), it is not surprising that Russian participants supported RWA and SDO more than Taiwanese participants in our study. Despite having relatives as immigrants may reduce Russian participants’ negative attitudes toward immigrants, Russian participants still showed more negative attitudes toward immigrants in comparison to Taiwanese participants, consistent with the group position’s interpretation (i.e., Russia, one of the powerful countries in the international context; Taiwan, one of the powerless countries in the international context).

In recent years, human societies have experienced dramatic changes due to their increasing mobility over time. People of different ethnicities, races, religions, nations, and social classes have opportunities to migrate and interact with others (Croucher et al., 2018); however, these interactions and contact may result in conflict. Our research provides a novel perspective in understanding how immigrants may be viewed in two societies relatively understudied. Our findings suggest that negative attitudes toward immigrants may vary according to the perceivers’ characteristics or experiences, be it ideology-based such as authoritarian beliefs or experience-based such as having relatives as migrants. Whereas ideology-based mechanisms are consistently observed in the two societies, experience-based mechanisms are culturally specific. In the ideology-based mechanisms, authoritarian beliefs serve to justify the negative attitudes toward immigrants; naïve dialecticism and incremental theories of the world could reduce the detrimental effects of authoritarian beliefs. In experience-based mechanisms, having relatives as migrants may allow individuals opportunities to learn about the predicament the immigrants are forced to endure. By understanding how people formulate their attitudes toward immigrants, perhaps a friendly and corroborating society could be formed.

## Data Availability Statement

The raw data supporting the conclusions of this article will be made available by the authors, without undue reservation.

## Ethics Statement

The studies involving human participants were reviewed and approved by the Research Ethics Committee of National Taiwan University (NTU-REC No.: 201805HS002). The patients/participants provided their written informed consent to participate in this study.

## Author Contributions

I-CL, TP, and MS developed the research ideas and materials together, collected the data in own country (I-CL in Taiwan, TP and MS in Russia), and revised the manuscript. I-CL conducted the data analysis and drafted the manuscript. TP drafted the parts of the manuscript pertaining to Russia. All the authors contributed to the article and approved the submitted version.

## Conflict of Interest

The authors declare that the research was conducted in the absence of any commercial or financial relationships that could be construed as a potential conflict of interest.

## Appendix

### Culturally Equivalent Items in Russia and Taiwan

#### Dialectical Self Scale (Spencer-Rodgers et al., 2015, Original Item Numbers: 5, 6, 7, 16, 19, 21, 23, 26, 30, 32)

(1) I often change the way I am, depending on who I am with.

(2) I often find that things will contradict each other.

(3) If I’ve made up my mind about something, I stick to it. (reversed)

(4) I am constantly changing and am different from one time to the next.

(5) I can never know for certain that any one thing is true.

(6) My core beliefs don’t change much over time. (reversed)

(7) I sometimes find that I am a different person by the evening than I was in the morning.

(8) I find that my world is relatively stable and consistent. (reversed)

(9) I have a hard time making up my mind about controversial issues.

(10) There are always two sides to everything, depending on how you look at it.

#### Social Dominance Orientation (Pratto et al., 2000; Ho et al., 2015)

(1) Some groups of people are simply inferior to other groups.

(2) An ideal society requires some groups to be on top and others to be on the bottom.

(3) It’s probably a good thing that certain groups are at the top and other groups are at the bottom.

(4) No one group should dominate in society. (reversed)

(5) We should not push for group equality.

(6) We shouldn’t try to guarantee that every group has the same quality of life.

(7) It is unjust to try to make groups equal.

(8) Group equality should be our ideal. (reversed)

#### Right-Wing Authoritarianism (Duckitt et al., 2010)

(1) What our country needs most is discipline, with everyone following our leaders in unity.

(2) Obedience and respect for authority are the most important virtues children should learn.

(3) Our country will be great if we show respect for authority and obey our leaders.

(4) The real keys to the “good life” are respect for authority and obedience to those who are in charge.

(5) The authorities should be obeyed because they are in the best position to know what is good for our country.

(6) This country will flourish if young people stop experimenting with drugs, alcohol, and sex, and pay more attention to family values.

(7) God’s laws about abortion, pornography, and marriage must be strictly followed before it is too late.

(8) The radical and sinful new ways of living and behaving of many young people may one day destroy our society.

(9) It is important that we preserve our traditional values and moral standards.

(10) The way things are going in this country, it’s going to take a lot of “strong medicine” to straighten out the troublemakers, criminals, and perverts.

(11) The situation in our country is getting so serious, the strongest methods would be justified if they eliminated the troublemakers and got us back to our true path.

(12) What our country really needs is a tough, harsh dose of law and order.
